# Pulmonary Malakoplakia by *Rhodococcus equi* in an HIV-Infected Patient in Mexico: A Case Report

**DOI:** 10.1155/2020/3131024

**Published:** 2020-04-06

**Authors:** Victor H. Ahumada, Ivan Ortiz-Monasterio, Jose L. Hernandez, Amy B. Peralta

**Affiliations:** Department of Research of Infectious Diseases, Instituto Nacional de Enfermedades Respiratorias Ismael Cosio Villegas, Mexico City, Mexico

## Abstract

**Background:**

*Rhodococcus equi*-related pulmonary malakoplakia is a rare condition with few reported cases; hereby, we present a case associated with advanced human immunodeficiency virus (HIV) infection, and thus far to our knowledge, the first report in Mexico. It is estimated that approximately 10% of the infections occur in immunocompetent patients, whereas the rest are immune deficient, targeting virtually any organ. Histologically, malakoplakia is characterized by the buildup of infiltrated inflammatory tissue as a consequence of the gathering of histiocytes embedded with concentric inclusions. The diagnosis relies on the cultures and the susceptibility testing as well as the pathologic findings compatible with the disease. *Case Presentation*. We present a 25-year-old male patient with persistent nonproductive cough for over a year and with weight loss, who comes to the emergency department with recent fever, swollen and tender lymph nodes, and hemoptysis. The patient gets diagnosed and treated for *Rhodococcus equi*-related pulmonary malakoplakia.

**Conclusion:**

Knowing the involvement of *Rhodococcus* in HIV is fundamental for the diagnosis and optimal treatment, which although unknown, a combination of antibiotics with intracellular penetration, on-time resection, and a proper immune reconstitution represents the best approach. Prognosis varies with mortality rates from 34% to 54%.

## 1. Background

Malakoplakia is a rare condition, histologically characterized by the buildup of infiltrated inflammatory tissue as a consequence of the gathering of histiocytes embedded with concentric inclusions known as Michaelis–Gutmann bodies, as well as the abnormal proliferation of macrophages within, forming a plaque known as xanthogranulomatous inflammation.

It is mostly appreciated as an infectious process that results in an acquired lysosomal impairment and a flawed elimination of ingested pathogens [1]. The resultant defective lysis is generally considered to be the cause of the inclusion bodies found microscopically [2]. A deficiency of 3′5′-guanidine monophosphate dehydrogenase has been proposed in [3, 4].

Although malakoplakia pseudotumor has been seen as a result of different kinds of infection such as *Mycobacterium tuberculosis*, *Pasteurella multocida*, *Escherichia coli*, *Tropheryma whipplei*, cytomegalovirus (CMV), and human simplex virus 2 (HSV2), the most commonly associated pathogen is thought to be *Rhodococcus* ssp. [17].

*Rhodococcus equi* previously known as *Corynebacterium equi* was first isolated in 1923, and not until 1967, it was reported in human beings for the first time. The microorganism is most commonly seen in a variety of land and water animals including, but not limited to, cattle, swine, reptiles, and birds. It can be found in any continent except for Antarctica, and it has the capacity to proliferate in freshwater, marine environments, and within the gastrointestinal system of arthropods. The means by which the infection can be acquired by humans is through inhaling the bacteria or direct inoculation to an open wound or a permeable mucosa that comes in contact with it, predominantly in immunocompromised hosts [15].

The vast majority of studies made in regards to *Rhodococcus* come from animal models, where several mechanisms of pathogenicity mediated by proteins of variable virulence have been found even within species and depending upon the host and its immunological status. In vitro observations have identified the presence of IL4 and INF-y and the formation on granulomas as part of the mechanism [15].

## 2. Case Presentation

A 25-year-old male patient from Tabasco, a tropical southern state in Mexico, presented with persistent nonproductive cough and weight loss of more than 10% its normal weight as chief complaints. He mentioned having the symptoms for a year, denying fever, shortness of breath, or other constitutional symptoms.

He allegedly started with whitish sputum, later mild hemoptysis (specks of blood) together with fever quantified above 102.2°F and resting dyspnea occurred until he reached for medical attention in a local hospital where a chest X-ray was taken, showing a total left atelectasis. The patient was later transferred to the National Institute of Respiratory Diseases (INER), a specialized referral center in Mexico city.

Additional medical history included MSM practices, occasionally with no protection. The patient denied the use of alcohol, illicit drugs, or nicotine, and he denied having followed the adult immunization schedule. He mentioned having no contact with domesticated or undomesticated animals whatsoever.

On admission, physical examination revealed a temperature of 102.7°F, a heart rate of 120 beats per minute, and a respiratory rate of 30 breathes per minute. The patient presented oral candidiasis and had multiple purple lesions with elevated borders affecting the skin and palate, with bilateral cervical nontender, indurated swollen lymph nodes. A hyperdynamic precordium was identified, with asymmetric chest expansion, diminished respiratory sounds, and fremitus as well as a flat sound on percussion.

His complete blood count on admission presented a normocytic-normochromic anemia of Hb10.4 g/dL (MCV80.9fL, MCH26.2pg, and RDW17.3%), Hct32.1%, leucocytes 6.500/*μ*L (lymphocytes 300/*μ*L), and platelets 183,000. Liver and renal function test appeared within normal rage, creatinine 0.6 mg/dl, AST 35 UI/L, ALT p26 UI/L, LDH 241 UI/L, ALP 93 UI/L, procalcitonin 0.84 *μ*g/L, and arterial blood gasses: pH 7.45, PCO2-26.4, PO2-51.6, HCO3-18, EB-(−)4.2, AG-21.1, SAT-82.7%, FiO2-27, and Lac-0.9.

The patient tested positive for HIV (p24Ag, anti-HIV and western blot), as well as for HHV8, with CD4 of 7 cells/*μ*L (2%) and a viral load of 126,916 copies/ml (log5.1) and tested negative for hepatitis B and C screening.

**Figure 1:**
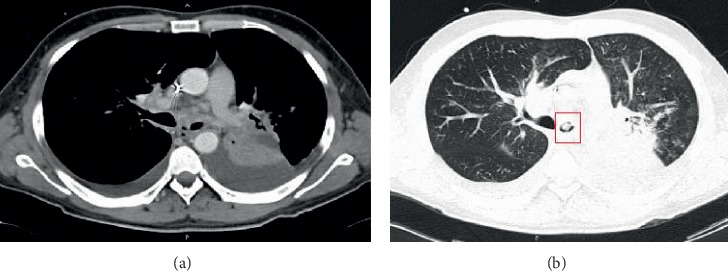
(a) Thorax CT showing a dense consolidation in the lower lung left lobe with mass aspect and pleural effusion. (b) Tumor obstructing 90% of the left main bronchus (red box).

The CT scan showed bilateral pleural effusion (Figure 1(a)) with an occupational lesion obstructing the left main bronchial lumen (Figure 1(b)) as well as a consolidation with air bronchogram in the left lung in segment 6. A diagnostic bronchoscopy was done, showing an emerging tumor in the left main bronchus with an 80% obstruction of its lumen with chronic inflammatory changes.

Blood cultures on admission turned positive for Gram-positive, catalase-positive, CAMP-positive coccobacillus compatible with *Rhodococcus equi* using a Biomerieux in vitro diagnostic technique. The antibiogram showed the microorganism to be TMP/SMX resistant, quinolone sensible, rifampicin sensible, and vancomycin sensible. The rest of the cultures were negative for other bacteria, mycobacteria, and fungus. Bronchioloalveolar lavage cultures turned positive for *Rhodococcus equi* too.

**Figure 2:**
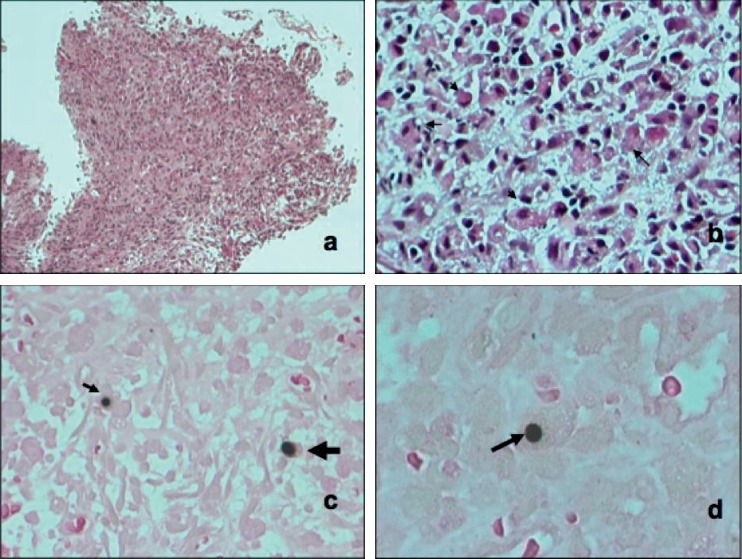
(a) Panoramic view of the lesion constituted by the stroma mixed with inflammatory cells. (b) A close up of the lesion, where several foamy histiocytes can be seen (arrows). (c, d) Some foamy histiocytes present intracytoplasmatic inclusion bodies with Von Kossa stain known as Michaelis–Gutmann bodies (arrows).

The histopathological report of the transbronchial biopsy (Figure 2) shows a cumulus of inflammatory cells with foamy histiocytes, some of which can be seen with intracytoplasmatic inclusions (Michaelis–Gutmann bodies) compatible with pulmonary malakoplakia. No neoplastic or necrotic tissue was reported. Five days later, a therapeutic bronchoscopy approach was made, with resection of tumor tissue freeing 95% of the bronchus lumen.

Besides, several skin and cervical lymph node biopsies were done resulting in Kaposi sarcoma, for which the patient received management with liposomal doxorubicin.

Two weeks after admission, the patient started HAART with the EFV/TDF/TFC regimen.

A combination with vancomycin 1 gr IV b.i.d and rifampicin 600 mg PO q.d. antibiotic scheme was used for 3 weeks and then was changed to ciprofloxacin 750 mg PO b.i.d. and rifampicin for 3 months more, given the chosen scheme. At the time of this report, the patient showed a fine recovery from the malakoplakia with full radiologic and symptomatic remission and a viral control at 6 months with considerable adherence to treatment. Thus far, no other intervention was required and the patient referred going back to his normal lifestyle.

## 3. Discussion

Even though there are few reported cases, therefore lack of research, it is estimated that approximately 10% of the infections occur in immunocompetent patients, whereas the rest are immune deficient, mainly patients with HIV [17].

Therefore, the majority of infections has something to do with patients with defects in cell-mediated immunity, with a history of contact with farm animals [2].

The key features depend mainly upon the immune state of the host, as well as the inoculum that the subject comes in contact with, as well as the risk factors involved. Symptoms can vary from those resembling cavitary pneumonia with hilar lymphadenopathy to that of intraluminal mass [5], which in the image studies may appear as so, or just as plain consolidation [17]. The presentation is variable, and two of the most common infectious sites are the lung and the kidney although it could affect virtually every organ varying from nodules to abscess to bacteremia [15].

When the infection occurs in the lungs, 40% of the cases in immunocompromised hosts [6], the disease normally takes a subacute progressive course characterized by cough, pleurisy, fever, and often constitutional symptoms that could follow concurrent bacteremia and disseminated disease.

The diagnosis, however, relies on the cultures and the susceptibility testing as well as the pathologic findings compatible with the disease, typically necrotic with dense histiocytic infiltrates with an eosinophilic, granular cytoplasm and intrahistiocytic coccobacilli. Serologic assays have not been clinically validated and are not commercially available [15].

Radiographic presentation of the infection most of the times appear to be cavitary upper lobe pneumonia, frequently misdiagnosed as pulmonary tuberculosis [2, 7]. Less frequently, rhodococcal infiltrations look like neoplasm; however, when this situation is encountered, it can easily be confused not only with lung cancer but also with mediastinal, costal, or vertebral. Differential diagnostics should always be taken into consideration [8]. The mortality rate ranges from 34% to 54%, much higher than that observed for patients without malakoplakia [1].

Given the advent of the HIV epidemic, studies have been made regarding *Rhodococcus equi* infections in these people, showing that the main independent predictor factors of mortality were the absence of highly active antiretroviral therapy (HAART), extensive pulmonary disease (multilobar infiltrates), and the inappropriate choice of antibiotic treatment [9]. A summary of reported cases is included in Table 1.

The treatment for this condition varies in some of the articles published and recommendations. Nonetheless, it is of great importance to accurately establish the most efficient treatment in each case, with sensitivity profiles, in order to prevent and bypass all complication foreseen, given that the prognosis of this condition might worsen very easily. Generally, for the optimal treatment, although unknown, it is a combination of antibiotics with intracellular penetration, and sometimes prolong schemes with quinolones, rifampin, macrolides, tetracycline, or a combination for systemic disease, with adequate on-time surgical resection, as CD4 T helpers type 1 cells, are needed and frequently underperformed on HIV-infected individuals [4, 15, 18]. Other sources recommend, in addition to the abovementioned, imipenem and vancomycin initially intravenous followed by oral administration for at least 6 months for pulmonary, osteoarticular, and central nervous system infections [4]. Francesca Moretti *et al*. supported in 2002 that effective HAART therapy and a short course of antibiotic therapy including levofloxacin can resolve *Rhodococcus equi* pneumonia without surgery in spite of a slow immune reconstitution [18].

## 4. Conclusions

Given the low incidence of this disease, the clinician requires high skills and acuity for suspecting *Rhodococcus*-associated malakoplakia in all unresolved pneumonia cases with nonneoplastic lung tumoral mass since the course of the disease in immunocompromised patients could be fatal.

Specific goals in achieving healing and a successful treatment include three main aspects: (1) combined and prolonged use of sensitivity-proven antibiotics of intracellular penetration, (2) precise surgical evaluation for on-time resection for patients who do not respond adequately to antibiotic therapy, and (3) efficient immune reconstitution with the adequate use of antiretroviral therapy.

In this case, our patient had a satisfactory recovery under a treatment based on rifampicin and ciprofloxacin over a period of 6 months, plus an optimal virologic control with HAART.

*Rhodococcus*-associated pulmonary malakoplakia in an uwnusual presentation of an uncommonly reported disease; this evidence serves for the record in advance HIV infection. Up to this point, according to the literature revised, this is the first reported case in Mexico and one of the few around the world.

More information and research are needed in this topic since this paper is just limited to the current literature and based on the findings on one single patient.

## Figures and Tables

**Table 1:** *Rhodococcus equi*-associated pulmonary malakoplakia in HIV-infected patients.

Age/sex	Immune status	Location	Endobronchial appearance	Lung findings	Culture results	Histologic features	Outcome	Reference
15F	HIV/AIDS	Left main stem bronchus, near carina	Occlusive masses	Cavitary lesion, necrotizing pneumonia	*R. equi* (PBL)	Sheets of PAS-positive foamy macrophages filled with Gram-positive coccobacilli	Death	Fidvi [10]

32M	HIV/AIDS	RML bronchus	Occlusive mass	RML consolidation	*R. equi* (PBL, BAL, pseudotumor)	Histiocytes with associated lymphocytes, plasma cells, and neutrophils; malakoplakia-like features	RML lobectomy	Canfrere [11]

30F	HIV/AIDS	LUL bronchus, LUL	Partially occlusive endobronchial mass, lung mass	LUL mass lesion	*R. equi* (bronchial aspirate, BAL, tissue)	Sheets of histiocytes with eosinophilic, faintly granular cytoplasm	LUL lobectomy, resolution	Bishopric [12]

25M	HIV/AIDS	Left main stem bronchus	Partially occlusive mass	Cavitary left lower lobe lesion	*R. equi* (bronchial aspirate)	Sheets of epithelioid histiocytes	Resolution	Shapiro [13]

47M	HIV/AIDS	Trachea	Occlusive mass with erosion to esophagus	Cavitary RUL lesion	*R. equi* (laryngeal pseudotumor)	Sheets of histiocytes with eosinophilic, coarsely granular cytoplasm	Persistence requiring tracheostom	Akilesh [14]

30M	HIV/AIDS	RML bronchus	Occlusive mass	RUL nodular lesion, right hilar lymphadenopathy	*R. equi* (BAL, blood)	Bland cells with abundant cytoplasm (CD68+), histiocytes with intracellular Gram-positive coccobacilli	Loss of follow-up	Krieg [15]

35M	HIV/AIDS	RLL bronchus	Bronchial tumor	RLL abscessed tumor with central necrosis	*R. equi* (sputum, BAL, lung biopsy)	Histiocytic infiltrate with intracellular inclusions (Michaelis–Gutmann bodies)	Resolution	Romero [16]

36F	HIV/AIDS	Multiple billateral lesions		Cavitary lesions	*R. equi* (fine needle aspirate culture)	Clusters of granular histiocytes with numerous intracytoplasmic and extracytoplasmic M-G bodies	Resolution	Sughayer [17]

RUL: right upper lobe, RML right middle lobe, RLL: right lower lobe, LUL: left upper lobe, PBL: peripheral blood, BAL: bronchioloalveolar lavage, MAI: *Mycobacterium avium* intracellulare complex, and ND: not done.

## Abbreviations

HIV: Human immunodeficiency virus
CMV: Cytomegalovirus
HSV2: Human simplex virus 2
INER: Instituto Nacional de Enfermedades Respiratorias
MSM: Men who have sex with men
HHV8: Human herpes virus 8
TMP/SMX: Trimethoprim sulfamethoxazole
HAART: Highly active antiretroviral therapy
PMHx: Past medical history
CC: Chief complaint
Ev: Evolution
Labs: Laboratory findings
Rad: Radiology findings
Dx: Diagnosis
Tx: Treatment.
